# A case of abnormal hypertension and Takotsubo syndrome caused by adrenal hemostasis using an electric scalpel: a case report

**DOI:** 10.1186/s40981-025-00791-y

**Published:** 2025-05-16

**Authors:** Tsutomu Suzuki, Naoko Kubo, Kei Kamiutsuri

**Affiliations:** Department of Anesthesiology, Rinku General Medical Center, Rinku Ourai Kita 2-23, Izumisanoshi, Osaka 598-8577 Japan

**Keywords:** Intraoperative hypertension, Adrenal gland, Takotsubo syndrome

## Abstract

**Background:**

Although intraoperative adrenal hemostasis by cauterization can cause abnormal hypertension, hemodynamic condition is usually normalized in a few minutes without any postoperative complications. We present a rare case of abnormal hypertension caused by adrenal hemostasis using an electric scalpel, which resulted in cardiac dysfunction: Takotsubo syndrome.

**Case presentation:**

A 74-year-old woman received open hepatectomy for a hepatic tumor. During adrenal electrocauterization, abnormal hypertension and tachycardia suddenly occurred. Although the blood pressure returned to the baseline in a few minutes by nicardipine and landiolol, postoperative echocardiography revealed apical hypokinesis and basal hyperkinesis of the left ventricular wall with a decreased ejection fraction of 50%. Along with no coronary artery stenosis by CT angiography, a diagnosis of Takotsubo syndrome was made. Postoperative course was uneventful; ejection fraction increased to 69% with no obvious left ventricular wall asynergy at 1-month postoperative follow-up.

**Conclusions:**

Adrenal cauterization during surgery may cause abnormal hypertension by release of excessive catecholamines, and potentially lead to Takotsubo syndrome. Anesthesiologists should be prepared to respond quickly to any unexpected changes in hemodynamics.

## Background

Hypertensive crises are induced by excessive catecholamine release following adrenal stimulation by electrocauterization or radiofrequency during surgery [1–3]. Although hemodynamic changes are usually controlled in a short period with no postoperative complications, they may lead to hypertensive emergency with end-organ damage.

Takotsubo syndrome is characterized by a temporary wall motion abnormality of the left ventricle [4], and sympathetic overdrive with increased catecholamines is one of the etiologies of this syndrome [5]. Although Takotsubo syndrome induced by hypertensive crisis is reported in patients with pheochromocytoma [6], there are few reports of this syndrome following abnormal hypertension by intraoperative electrocauterization. We present a rare case of Takotsubo syndrome following abnormal hypertension caused by adrenal hemostasis with electrocauterization.

## Case presentation

A 74-year-old woman (height: 150 cm, weight: 52.9 kg) was scheduled to undergo open right lobe hepatectomy for a hepatic tumor. She had a history of total hysterectomy with bilateral adnexectomy for a left ovarian tumor under general anesthesia without perioperative hypertensive episode 1 month prior. Her oral medication was powdered senna leaf only. The abdominal CT scan revealed no obvious adrenal tumor. Blood tests and electrocardiogram results were normal. Transthoracic echocardiography showed no significant abnormalities in the heart valves or wall motion, with an ejection fraction of 60%.

**Fig. 1:**
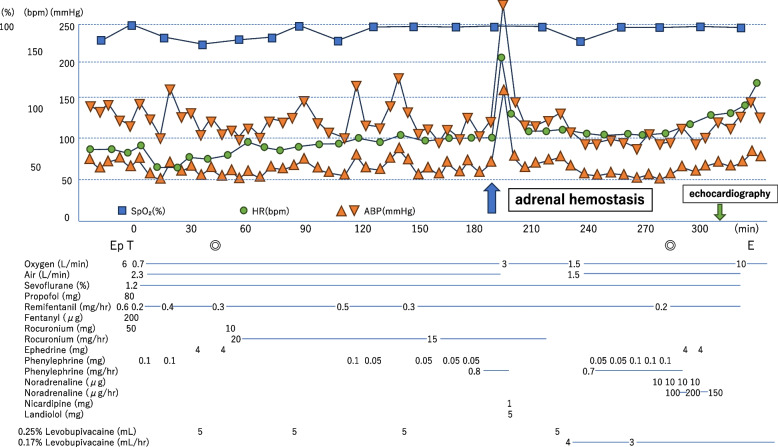
Anesthesia record. Heart rate increased to > 150 bpm, followed by a rapid rise in systolic blood pressure to 280 mmHg by adrenal electrocauterization two and a half hours after starting surgery. Blood pressure returned to the baseline level within 10 min by nicardipine 1 mg and landiolol 5 mg. Hypotension was managed by administering phenylephrine and noradrenaline until the end of anesthesia. Upon leaving the operating room, the patient was stable without the need for vasopressors or inotropic agents. SpO2, percutaneous oxygen saturation; HR, heart rate; ABP, arterial blood pressure; Ep, epidural anesthesia; T, tracheal intubation; E, extubation; ◎, start and end of the surgery

When she arrived at the operating room, her vital signs were stable, with a blood pressure of 139/64 mmHg, a heart rate of 59 bpm, and an SpO_2_ of 98% on room air. After insertion of an epidural catheter through the Th8-9 intervertebral space, general anesthesia was induced using 100 μg of fentanyl, 80 mg of propofol, and 50 mg of rocuronium, and maintained with sevoflurane, remifentanil, and rocuronium. The left radial artery was cannulated for blood pressure monitoring, and a central venous catheter was placed in the right internal jugular vein. Intraoperatively, 5 mL of 0.25% levobupivacaine was administered via the epidural catheter in four separate bolus doses. Intraoperative hypotension was managed by bolus phenylephrine, and systolic blood pressure fluctuated between 80 and 110 mmHg. The heart rate remained stable at 70–90 bpm (Fig. 1).

**Fig. 2:**
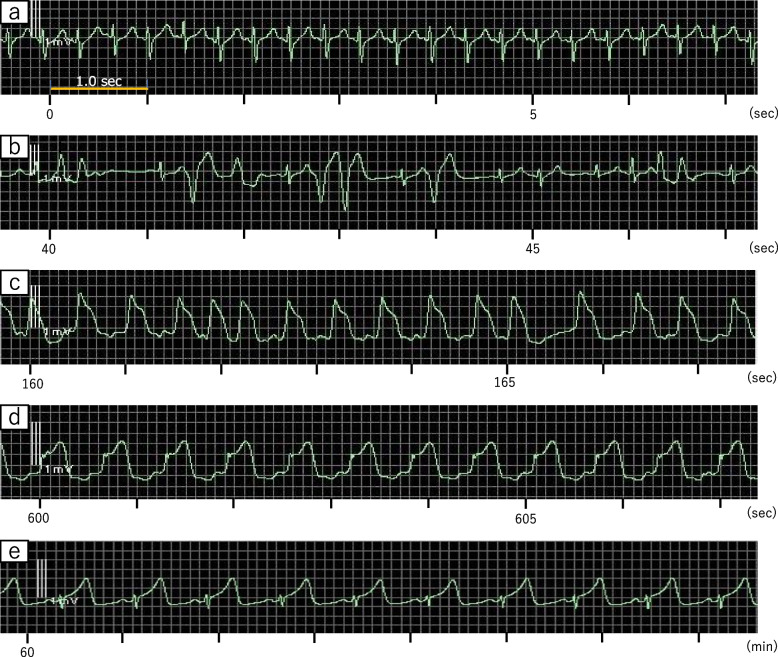
ECG (II lead) after hypertensive crisis. Shortly after an increase of heart rate (**a**), multifocal ventricular extrasystole appeared (**b**), followed by elevation of ST segments in 2 min (**c**), which persisted 10 min later (**d**). Elevation of ST segments persisted 60 min later (**e**)

Two and a half hours after starting surgery, heart rate abruptly surged to > 150 bpm, with a rapid rise in systolic blood pressure to 280 mmHg during electrocauterization for parenchymal bleeding in the adrenal gland after resection of the liver (Fig. 2a). ECG changed to multifocal ventricular extrasystole (Fig. 2b), which progressed to arrhythmia with elevated ST segments within 2 min (Fig. 2c). Although blood pressure returned to its baseline level within a few minutes after administrating nicardipine 1 mg and landiolol 5 mg, ST segment elevation remained in the ECG monitor (Fig. 2d, e). Blood pressure decreased to approximately 80–90/40 mmHg with stable heart rate around 80 bpm approximately 30 min after the hypertensive episode and was managed by continuous infusion of phenylephrine and noradrenaline until the end of surgery. Planned surgery was completed approximately 1.5 h later; duration of surgery was 4 h 7 min and total blood loss was 280 mL. The patient left the operating room after extubation with stable hemodynamic and respiratory conditions (Fig. 1), with no abnormal neurological findings.

**Fig. 3:**
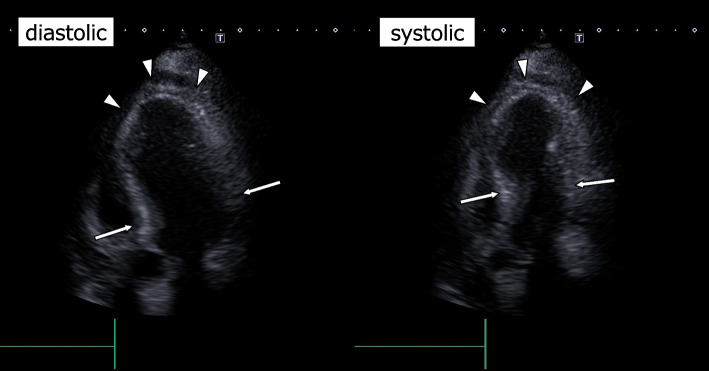
The postoperative transthoracic echocardiography of apical four chamber view. The echocardiography revealed apical hypokinesis (white arrowheads) and basal hyperkinesis (white arrows) of the left ventricular wall

Postoperative transthoracic echocardiography 20 min after the surgery revealed apical hypokinesis and basal hyperkinesis of the left ventricular wall with an ejection fraction of 50% (Fig. 3). The blood pressure was 120/60 mmHg with a heart rate of 90 bpm. In combination with no stenosis in the coronary arteries revealed by postoperative CT angiography, her condition was diagnosed as Takotsubo syndrome according to the criteria [4]. With no episodes of arrhythmias or left ventricular hypokinesis, the patient was discharged from the intensive care unit on the first postoperative day. There were no severe arrhythmias or symptoms of heart failure during hospitalization, and she was discharged on the 13th day after surgery. Transthoracic echocardiography at the 1-month postoperative follow-up showed an improvement in ejection fraction to 69% and no obvious left ventricular wall asynergy.

## Discussion

We experienced a case of hypertensive crisis during adrenal hemostasis using an electric scalpel, which resulted in Takotsubo syndrome.

The causes of sudden abnormal intraoperative hypertension include inadequate anesthesia, hypoxia, hypercarbia, and pre-existing diseases such as essential hypertension and pheochromocytoma [7]. In this case, there was no history of hypertension or heart disease, and peripheral oxygen saturation and end-tidal cardon dioxide tension were within normal limits during anesthesia. Although preoperative adrenal hormone levels were not measured, there were no adrenal tumors, ruling out the possibility of pre-existing diseases or inadequate anesthesia for this episode.

Fransson et al. reported radiofrequency ablation of normal adrenal tissue induced remarkable hypertension by an increase of blood noradrenaline levels in dogs [8]. In that report, systolic blood pressure increased to more than 200 mmHg, and blood norepinephrine levels were 39 times higher than baseline levels. This is similar to a condition caused by intraoperative manipulation of pheochromocytoma. Because catecholamine excess by pheochromocytoma is one of the causes of Takotsubo syndrome [6, 9], hypertensive crisis would be the cause of Takotsubo syndrome in this case.

To manage the abnormal hypertension caused by the release of catecholamines from the adrenal gland, we immediately ceased hemostatic manipulation and administered calcium channel blockers, as is standard in the treatment of pheochromocytoma [10], followed by beta-blockers, although there is no established protocol for dealing with this condition.

The patient was closely monitored for the onset of heart failure symptoms or potentially life-threatening arrhythmias. Fortunately, no severe complications arose, and the patient was subsequently discharged home. Takotsubo syndrome is a well-known form of reversible acute heart failure; however, it was demonstrated that in-hospital complications were comparable between patients with Takotsubo syndrome and those with acute coronary syndrome [11]. As a therapeutic strategy, the use of angiotensin-converting enzyme inhibitors or angiotensin-receptor blockers was associated with improved survival at 1 year [11].

When adrenal hemostasis is performed, it is essential to be aware of the potential risk of developing abnormal hypertension. In such cases, an arterial catheter should be placed and blood pressure should be continuously monitored. Furthermore, any impact on cardiac function may complicate intraoperative circulatory control. Therefore, adrenal hemostatic manipulation without cauterization, such as compression hemostasis and suturing, is necessary to prevent catecholamine release.

## Conclusions

Cauterization of the adrenal glands can lead to the release of catecholamines, resulting in abnormal hypertension and Takotsubo syndrome. Anesthesiologists should be aware of the risks associated with adrenal hemostasis and respond promptly to any sudden changes in hemodynamics.

## Data Availability

The datasets used during the current study are available from the corresponding author on reasonable request.

## Abbreviations

CT: Computed tomography
SpO_2_: Percutaneous oxygen saturation
ECG: Electrocardiogram
